# Identifying reference genes with stable expression from high throughput sequence data

**DOI:** 10.3389/fmicb.2012.00385

**Published:** 2012-11-09

**Authors:** Harriet Alexander, Bethany D. Jenkins, Tatiana A. Rynearson, Mak A. Saito, Melissa L. Mercier, Sonya T. Dyhrman

**Affiliations:** ^1^MIT-WHOI Joint Program in Oceanography/Applied Ocean Science and EngineeringCambridge, MA, USA; ^2^Biology Department, Woods Hole Oceanographic InstitutionWoods Hole, MA, USA; ^3^Graduate School of Oceanography, University of Rhode IslandNarragansett, RI, USA; ^4^Department of Cell and Molecular Biology, University of Rhode IslandKingston, RI, USA; ^5^Department of Marine Chemistry and Geochemistry, Woods Hole Oceanographic InstitutionWoods Hole, MA, USA

**Keywords:** *Thalassiosira pseudonana*, diatom, phytoplankton, housekeeping genes, RT-qPCR, transcriptome, relative gene expression, reference gene

## Abstract

Genes that are constitutively expressed across multiple environmental stimuli are crucial to quantifying differentially expressed genes, particularly when employing quantitative reverse transcriptase polymerase chain reaction (RT-qPCR) assays. However, the identification of these potential reference genes in non-model organisms is challenging and is often guided by expression patterns in distantly related organisms. Here, transcriptome datasets from the diatom *Thalassiosira pseudonana* grown under replete, phosphorus-limited, iron-limited, and phosphorus and iron co-limited nutrient regimes were analyzed through literature-based searches for homologous reference genes, *k*-means clustering, and analysis of sequence counts (ASC) to identify putative reference genes. A total of 9759 genes were identified and screened for stable expression. Literature-based searches surveyed 18 generally accepted reference genes, revealing 101 homologs in *T. pseudonana* with variable expression and a wide range of mean tags per million. *k*-means analysis parsed the whole transcriptome into 15 clusters. The two most stable clusters contained 709 genes, but still had distinct patterns in expression. ASC analyses identified 179 genes that were stably expressed (posterior probability < 0.1 for 1.25 fold change). Genes known to have a stable expression pattern across the test treatments, like actin, were identified in this pool of 179 candidate genes. ASC can be employed on data without biological replicates and was more robust than the *k*-means approach in isolating genes with stable expression. The intersection of the genes identified through ASC with commonly used reference genes from the literature suggests that actin and ubiquitin ligase may be useful reference genes for *T. pseudonana* and potentially other diatoms. With the wealth of transcriptome sequence data becoming available, ASC can be easily applied to transcriptome datasets from other phytoplankton to identify reference genes.

## Introduction

Quantitative reverse transcriptase polymerase chain reaction (RT-qPCR) facilitates rapid, accurate, high-throughput analyses of gene expression, greatly enhancing and expanding molecular biological studies in a variety of organisms. This method has moved beyond the realm of model organisms (Adib et al., 2004; Antonov et al., 2005; Caldwell et al., 2005; Marionneau et al., 2005; Flatt et al., 2008) to be employed for the examination of ecological and physiological characteristics of marine microbes in both culture and the environment (Zehr and Turner, 2001; Nicot et al., 2005; Maldonado et al., 2006; Kustka et al., 2007; Allen et al., 2008; Mock et al., 2008; Lin et al., 2009; Zhao et al., 2009; Whitney et al., 2011; Wurch et al., 2011). There are two primary methods of gene expression analysis for single genes: (1) absolute quantification, whereby the copy number of a gene is determined through comparison of the PCR signal to a standard curve, and (2) relative gene expression, in which the expression of the gene of interest is determined through comparison to a reference gene (or internal control gene), often employing the 2^−ΔΔ*CT*^ method (Livak and Schmittgen, 2001; Pfaffl, 2001; Schmittgen and Livak, 2008).

Inherent in the 2^−ΔΔ*CT*^ method is the selection of a reference, or “housekeeping,” gene to act as an endogenous control. Ideally, the expression levels of the selected reference gene should remain stable across the treatments being examined. Genes like GAPDH, actin, and rRNA are often targeted as possible reference genes and tested for consistency in expression across treatments (Vandesompele et al., 2002; Pfaffl et al., 2004; Radonic et al., 2004). However, both Czechowski et al. (2005) and de Jonge et al. (2007) demonstrated that canonical reference genes were often extremely differentially regulated. In fact, de Jonge et al. (2007) noted that commonly used reference genes were not represented in the 50 most stably expressed genes in the human genome. Results from RT-qPCR studies using improper reference genes (e.g., genes that are not constitutively expressed) can be significantly different from results obtained with a proper reference gene (Dheda et al., 2005; Lanoix et al., 2012). Considering that previously established reference genes were not among the mostly stably expressed genes in model organisms, basing the selection of candidate genes for non-model organisms solely upon known reference genes may not prove the best method (Czechowski et al., 2005; de Jonge et al., 2007).

Application of RT-qPCR has proven particularly fruitful in the study of marine phytoplankton, illuminating transcriptional responses to physical stressors (Rosic et al., 2010a,b), nutrient limitation (Davis et al., 2006; Moseley et al., 2006; Berg et al., 2008; Davis and Palenik, 2008; Stuart et al., 2009; Whitney et al., 2011; Wurch et al., 2011; Bender et al., 2012), and the diel cycle (Whitney et al., 2011; Bender et al., 2012), as well as highlighting the modulation and activity of many metabolic pathways (Moseley et al., 2006; McGinn and Morel, 2008a; Mock et al., 2008; Bender et al., 2012). The success of these studies hinged upon the selection of a stably expressed reference gene. While there is often extensive literature characterizing the dynamics of suites of genes expressed under different conditions in studies of model organisms, similar breadth is lacking for non-model organisms, such as marine phytoplankton. With few genome sequences available, the selection of reference genes for eukaryotic phytoplankton is a challenge, and researchers must often choose candidate genes [e.g., actin (Nicot et al., 2005), GAPDH (Czechowski et al., 2005)] based on the literature from model organisms that are distantly related to the study organism. Selecting and validating potential reference genes is a difficult task that consequently slows the development and application of targeted gene expression studies for phytoplankton.

Screening the wealth of sequence data produced by modern ultra-high-throughput sequencing technologies may advance and broaden the search for candidate reference genes in non-model organisms. This is particularly true of transcriptome datasets whereby genes with stable expression can be identified between treatment conditions. Two statistical techniques, *k*-means clustering (Hartigan and Wong, 1979) and analysis of sequence counts (ASC) (Wu et al., 2010), usually used to investigate patterns of differential expression in transcriptome datasets, show promise in this regard. The *k*-means algorithm is a partition-based, non-hierarchical clustering method, which divides sequence tags into the specified *k*-number of clusters, while minimizing the intra-cluster spread based on the specified distance metric (Hartigan and Wong, 1979; Tavazoie et al., 1999; Gerstein and Jansen, 2000; Quackenbush, 2001; D'haeseleer, 2005). ASC is a novel empirical Bayes method (estimating the prior distribution from the data, itself) to detect differential gene expression generated from quantifiable gene expression counts (as generated by Illumina Digital Gene Expression tag profiling, RNA-seq or similar high-throughput sequencing technologies) (Wu et al., 2010). When applied to transcriptome data these tools cannot only be used to identify genes with differential expression, they can be used to identify genes with highly stable expression patterns.

Here, literature-based searches, *k*-means clustering, and ASC are compared as tools for reference gene selection using a transcript sequence dataset collected from the centric diatom *Thalassiosira pseudonana*, grown under nutrient replete, phosphorus-limited (P-limited), iron-limited (Fe-limited), and phosphorus and iron co-limited (Co-limited) treatments.

## Materials and methods

### Culturing and transcriptome data collection

**Figure A1:**
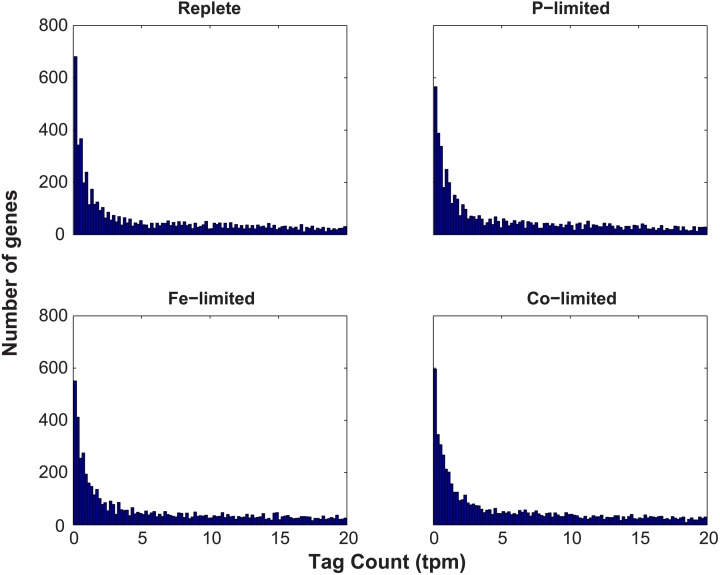
**Histogram analysis of the distribution of normalized tag counts (tpm) for each gene across each of the four treatments (Replete, P-limited, Fe-limited, and Co-limited).** The abundance of normalized tag counts (tpm) was assessed, tallying the total number of genes with a given tag count. Only tag counts less than 20 are depicted to aid the visualization of the inflection in the data at 2.5 tpm.

Axenic *T. pseudonana* CCMP 1335 was grown at 14°C under 24 h light (120 μmol photons m^−2^ s^−1^) after Dyhrman et al. (2012) in f/2 plus silica chelated media made from surface Sargasso Sea water. Nitrate, silica, vitamins, and trace metals were at f/2 concentrations (Guillard, 1975), while iron and phosphate were modified across treatments. In brief, triplicate cultures of replete (36 μM PO_4_, 400 nM Fe), P-limited (0.4 μM PO_4_, 400 nM Fe), Fe-limited (36 μM PO_4_, 40 nM Fe), and Co-limited (0.4 μM PO_4_, 40 nM Fe) treatments were harvested when growth deviated from the replete control. Growth was monitored by cell counts. Biomass was harvested onto 0.2 μm filters and flash frozen in liquid nitrogen and total RNA was extracted as described in Dyhrman et al. (2012). Tag-seq sequencing of the transcriptome was performed by Illumina with a polyA selection and NlaIII digestion, resulting in 21 bp sequence reads or tags (Dyhrman et al., 2012). Libraries were of varied sizes as follows: replete (~12 million), P-limited (~13 million), Fe-limited (~23 million), and Co-limited (~26 million). Tags were mapped to gene models (predicted protein coding regions) with a pipeline designed by Genesifter Inc., requiring 100% identity and covering 9759 genes. Tag counts within a gene were pooled and normalized to the size of the library, with resulting data expressed in tags per million (tpm). Genes with normalized tag counts less than 2.5 tpm for each of the four treatments were excluded (Figure A1), leaving 7380 genes in the analysis. The data discussed in this publication have been deposited in NCBI's Gene Expression Omnibus (GEO) (Edgar, 2002) and are accessible through GEO Series accession number GSE40509 (http://www.ncbi.nlm.nih.gov/geo/query/acc.cgi?acc=GSE40509).

### Reference gene identification

The current, relevant literature from algae and plant-based studies was queried for reference genes used as endogenous controls for relative gene expression assays. Stably expressed genes reported in the literature were compared using BLASTn (Altschul et al., 1997) against the *T. pseudonana* genome in NCBI (AAFD00000000.2) to find homologs (e-value <1.0 e-1). A loose e-value cutoff was used to be inclusive and enhance our collection of all potential reference gene candidates. In addition, the Eukaryotic Orthologous Group (KOG) definitions for the genes found via BLAST were identified, and subsequent genes located in the KOG definition families were included in the analysis.

For the *k*-means analysis, tag counts from the four treatments corresponding to the 7380 genes with reads greater than 2.5 tpm were clustered using the *k*-means algorithm under the Pearson correlation coefficient. The distance was measured with a Pearson correlation as it has been found to perform as well or better than other similar distance measures for non-ratio or count-based data (Gibbons and Roth, 2002), such as the *T. pseudonana* transcriptome dataset. The number of clusters (*k*) was determined via a figure of merit (FOM) estimation, which is an approximation of the predictive power of the clustering method (Yeung et al., 2001). FOM analysis was performed by predicting the FOM value for values of *k* ranging from *k* = 1 (one cluster) to *k* = 50 (50 clusters). The FOM value decreases as the within-cluster similarity increases, thus the FOM value was minimized to determine the optimal *k*-value. All clustering analyses were performed using the multiexperiment viewer (MeV) version 4.7 (Saeed et al., 2003, 2006). Possible reference gene targets were identified by isolating clusters of genes that exhibited similarly stable expression patterns across the four treatments.

Using ASC, the statistical significance of an observed fold change was determined in pairwise comparisons between each of the limited treatments and the replete control. The posterior probability (post-*p*) was calculated by computing the posterior mean of the log ratio of proportions over each of the P-limited, Fe-limited, and Co-limited treatments relative to the replete treatment for a fold change of 1.10, 1.25, and 1.50. Possible constitutively expressed genes were identified by selecting genes for which the post-*p* of each of the nutrient-limited treatments relative to the replete treatment for each of the fold change values was less than a specified cut-off. Posterior probability cutoffs between 0.01 and 0.20 were assessed across each of the fold changes (Table 1). Ultimately, a post-*p* of 0.10 was selected for further analyses (meaning that genes selected had less than a 10% chance of having the specified fold change between treatments), for it yielded genes across all of the fold change bins examined and demonstrated a broader range of mean normalized tag counts than seen for a post-*p* of 0.05 or 0.01. All ASC analyses were made using ASC 0.1.5 in R (http://R-project.org).

**Table 1:** **Gene counts for the fold change bins of 1.50, 1.25, and 1.10 across posterior probability cutoffs ranging from 0.01 to 0.20**.

	**Fold change**								
	**1.50**			**1.25**			**1.10**		
	**Number of genes**	**Minimum mean normalized tag count (tpm)**	**Maximum mean normalized tag count (tpm)**	**Number of genes**	**Minimum mean normalized tag count (tpm)**	**Maximum mean normalized tag count (tpm)**	**Number of genes**	**Minimum mean normalized tag count (tpm)**	**Maximum mean normalized tag count (tpm)**
**POSTERIOR PROBABILITY**									
post-*p* < 0.2	1649	2.11	1802.38	312	2.83	1281.15	8	20	176.63
post-*p* < 0.1	1375	2.22	1802.38	179	7.06	1281.15	2	51.81	105.73
post-*p* < 0.05	1127	2.83	1802.38	122	20	1281.15	1	105.73	105.73
post-*p* < 0.01	801	5.69	1802.38	62	20	1281.15	0	NA	NA

## Results

Transcript sequence data was generated from *T. pseudonana* CCMP 1335, grown in four different treatments (replete, P-limited, Fe-limited, and Co-limited). Potential reference genes were identified through (1) querying the data to identify expression of common reference genes based on literature searches, (2) a pattern-driven analysis using *k*-means clustering (Hartigan and Wong, 1979), and (3) a quantitative analysis based the probability of fold change using ASC.

**Figure 1:**
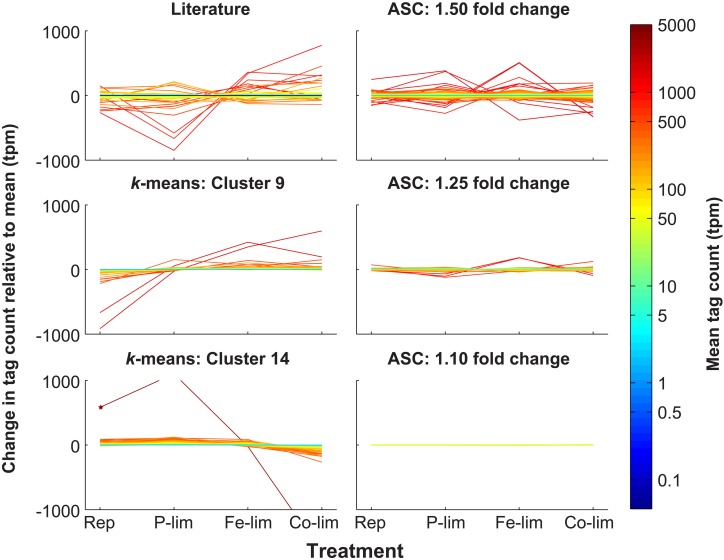
**Expression patterns of putative reference genes identified through literature-based searches, *k*-means clustering, and ASC analysis.** Through literature-based searches, a total of 101 genes homologous to reference genes from previous studies on plants and algae were identified in *T. pseudonana* and plotted to indicate deviation and mean tpm (Literature). *k*-means clustering was applied to the 7380 genes and Cluster 9 (243 genes) and Cluster 14 (466 genes) possessed the genes with the most stable expression pattern across the four treatments. Genes from these clusters are plotted to indicate deviation and mean tpm (*k*-means: Cluster 9; *k*-means: Cluster 14). ASC was used to assess statistical significance (post-*p* < 0.1) of fold changes of 1.10, 1.25, and 1.50 for each treatment relative to the replete control. Genes from these fold change bins are plotted to indicate deviation and mean tpm (ASC: 1.50 fold change; ASC: 1.25 fold change; ASC 1.10 fold change). For a fold change of 1.10, two genes, both hypothetical proteins, (NCBI: 7446346 and 7452192) passed the post-*p* < 0.1 cutoff, and represent the most stable genes based on the ASC analysis (**Data Sheet 3**). For each of the six classes of putative reference genes, tag counts were normalized to total library size (in tpm) and are plotted relative to the mean for each of the four treatments: Rep, Replete; P-lim, P-limited; Fe-lim, Fe-limited; and Co-lim, Co-limited. The color of the line correlates to the mean normalized tag count. A star marks a gene (NCBI: 7451632) in Cluster 14 that is not on the scale of expression for P-limited (1104.7 tpm) and Co-limited (-1664.9 tpm) treatments.

Selection of reference genes often falls upon those used in previous relative expression studies. The literature was surveyed for RT-qPCR expression studies employing the 2^−ΔΔ*CT*^ method for the following algae and plants: *T. pseudonana* (Maldonado et al., 2006; McGinn and Morel, 2008a,b; Mock et al., 2008; Park et al., 2008; Carvalho and Lettieri, 2011; Whitney et al., 2011), *Thalassiosira weissflogii* (Davis et al., 2006; McGinn and Morel, 2008a; Park et al., 2008; Whitney et al., 2011), *Phaeodactylum tricornutum* (Siaut et al., 2007; McGinn and Morel, 2008a), *Emiliana huxleyi* (Bruhn et al., 2010; Richier et al., 2010), *Micromonas pusilla* (McDonald et al., 2010), *Chlamydomonas reinhardtii* (Moseley et al., 2006; Zhao et al., 2009), *Alexandrium* spp. (Lee et al., 2009; Moustafa et al., 2010), *Symbiodinium* sp. (Rosic et al., 2010a,b; Leggat et al., 2011), *Prorocentrum minimum* (Guo and Ki, 2011), *Aureococcus anophagefferens* (Berg et al., 2008; Wurch et al., 2011), *Solanum tuberosum* (Nicot et al., 2005), and *Arabidopsis thaliana* (Avonce et al., 2004). Results from the current literature survey yielded a list of 18 key reference genes frequently employed in the study of gene expression for eukaryotic phytoplankton and plants: actin, calmodulin, cyclin dependent kinase, cyclophilin, cytochrome *c*, G-protein beta subunit, ferric enterobactin binding periplasmic protein precursor, histones, elongation factors, GAPDH, heat shock protein 90, poly(A) polymerase, ribosomal protein large subunit, ribosomal protein small subunit, SAM, α-, β-, γ-tubulin, and ubiquitin conjugating enzymes (**Data Sheet 1**). It is important to note that as more reference genes are validated as stable, the selection of putative reference genes may expand. The 101 genes identified as homologous to these reference genes across the four treatments in *T. pseudonana* had variable expression patterns and a wide range of mean normalized counts (0.08–1087.8 tpm) (Figure 1). Genes within a specific gene family (e.g., the five actin genes) had different mean counts as well as variable coefficients of variation (CV), which is indicative of variable expression (**Data Sheet 1**). For example, ACT 1 (NCBI: 7449411) had a mean expression of 1024.1 tpm and a CV of only 12.3%, whereas ACT 5 (NCBI: 7445819) had a lower mean expression of 23.95 and a higher CV of 35.5% (**Data Sheet 1**).

**Figure 2:**
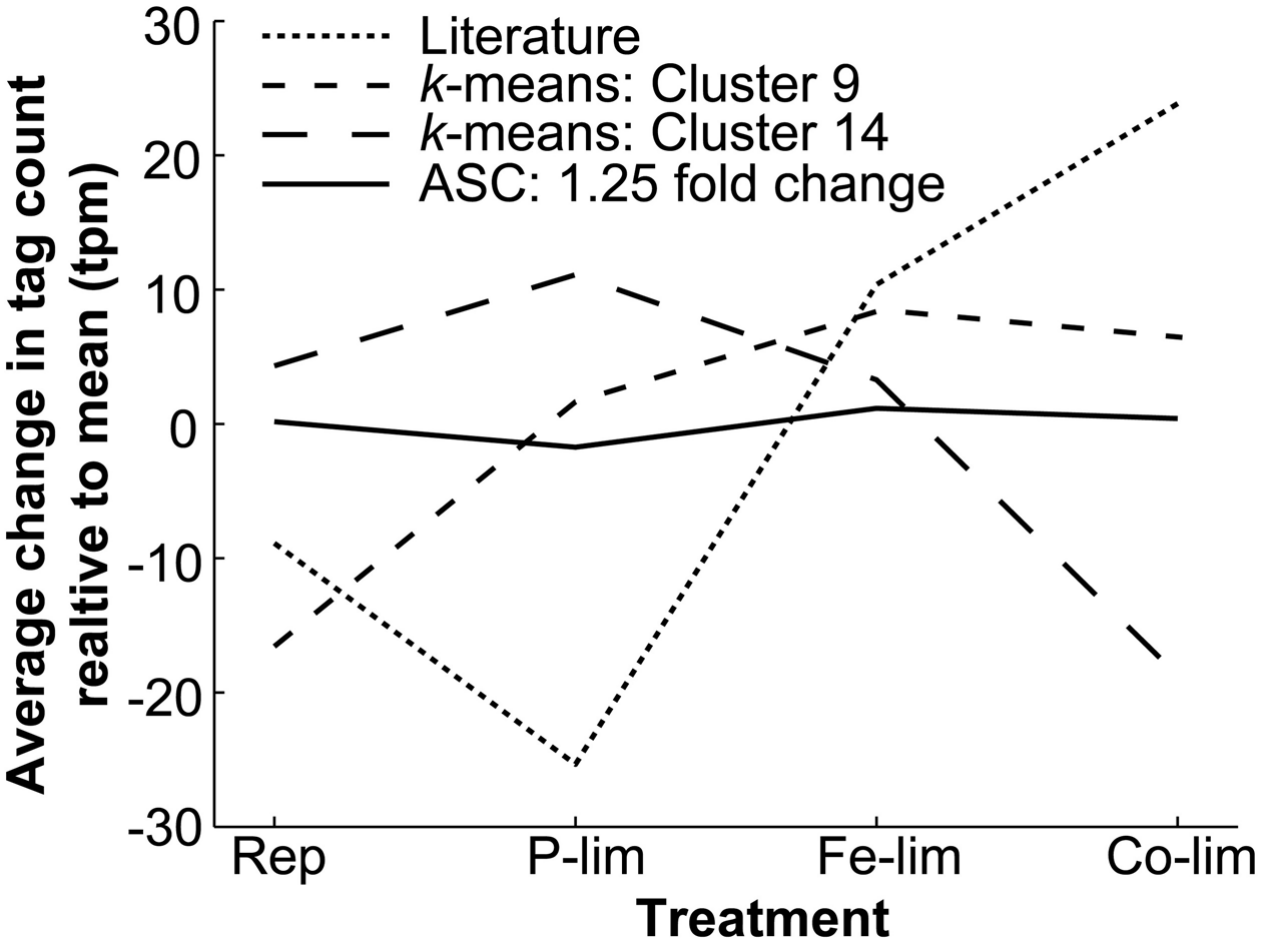
**Average deviation from the mean level of expression for all genes found with literature-based searches, *k*-means clustering, and ASC analysis of 1.25 fold change.** The average change in tag count from the mean expression (tpm) for all the genes identified through literature-based searches for genes homologous to known reference genes from the literature (*n* = 101), *k*-means clustering from Cluster 9 (*n* = 243) and Cluster 14 (*n* = 466), and ASC analysis identifying genes demonstrating a 1.25 fold change with a post-*p* < 0.1 (*n* = 179). The mean standard deviations for the four cases are as follows: Literature (92.62 tpm), Cluster 9 (41.66 tpm), Cluster 14 (43.12 tpm), and ASC (14.24 tpm). The mean tpm is plotted for the four treatments: Replete (Rep), P-limited (P-lim), Fe-limited (Fe-lim), and Co-limited (Co-lim).

**Figure A2:**
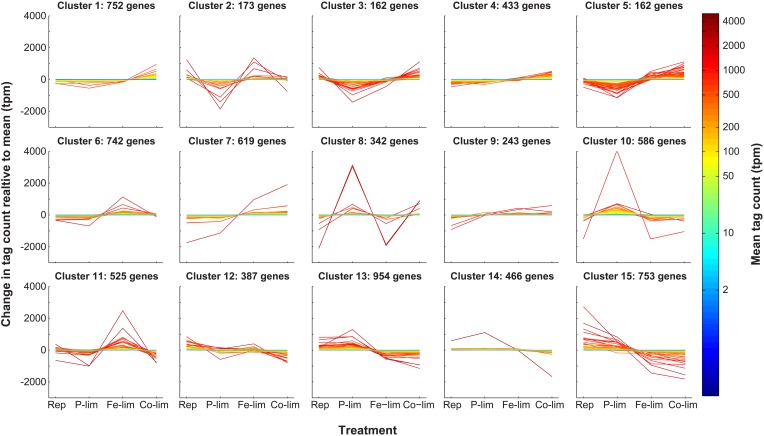
***k*-means clustering of normalized genes.** The 7380 genes that passed the 2.5 tpm cutoff were clustered into 15 clusters using the *k*-means algorithm under the Pearson correlation coefficient. Tag counts normalized to total library size (in tpm) for each gene are plotted relative to the mean (indicated by the color of the line) for each of the four treatments: Replete (Rep), P-limited (P-lim), Fe-limited (Fe-lim), and Co-limited (Co-lim).

The high-throughput transcript dataset was analyzed with *k*-means clustering. Prior to performing *k*-means cluster analysis, FOM optimization was run and found to be minimized at *k* = 15. Thus, *k*-means analysis was run under the Pearson correlation coefficient for *k* = 15, yielding 15 clusters, for which the intra-cluster variation was minimized (Figure A2). Of the 15 clusters produced (ranging in size from 162 to 954 genes), Cluster 4 (433 genes), Cluster 9 (243 genes), and Cluster 14 (466 genes) had candidate reference genes based on a low magnitude of change associated with the expression patterns in those clusters (Figure A2). However, Cluster 4 showed a clear pattern of differential regulation (down-regulated in the replete and up-regulated in the Co-limited), and as such it was not considered to be an optimal candidate cluster and was excluded from additional analyses. Both Cluster 9 and Cluster 14 consisted of genes with a wide range in mean tpm values (1.74–4191.91 tpm), with relatively small deviations from the mean value (Figure 1; **Data Sheet 2**), which stands in contrast to other clusters that had definite treatment driven expression patterns (Figure A2). Despite the relatively small deviations from the mean value, genes in Clusters 9 and 14 displayed both clear patterns of regulation, as demonstrated by the average change in tag count relative to the mean (Figure 2) and the presence of “outlier” genes with differential expression such as NCBI: 7451632, which was down-regulated in the Co-limited treatment for Cluster 14 (Figure 1; **Data Sheet 2**).

Adapting ASC to examine stable expression patterns, genes for which the post-*p* was less than 0.1 (e.g., had less than a 10% chance of equaling or exceeding the fold change cut-off) were plotted in three low fold change bins: 1.10, 1.25, and 1.50. A post-*p* of 0.1 was selected as it optimized the dataset for a wide range of mean gene expression values and provided coverage for each of the fold change bins examined (Table 1). The number of genes in each of the fold change bins increased with increasing value of fold change. For example, two genes passed the 1.10 cut-off, 179 genes passed the 1.25 cut-off, and 1375 genes passed the 1.50 cutoff. With the increase in the number of genes came an increase in the variation from the mean of the normalized tag counts (Figure 1; **Data Sheet 3**).

The bin with the 1.10 cutoff had two genes (NCBI: 7446346 and 7452192), which are both hypothetical proteins (Figure 1). A BLASTn search of 7446346 against the nr NCBI database yielded 69% identity over 251 base pairs (e-value, 1e-13) to a hypothetical protein (NCBI: CP000544.1) from *Halorhodospira halophila*, a salt-tolerant purple bacterium, and 69% identity over 232 base pairs (e-value, 1e-12) to a hypothetical protein (NCBI: CP001905.1) from *Thioalkalivibrio* sp. K90mix, also a salt-tolerant chemolithoautotrophic bacteria. BLASTp searches of 7452192 showed the highest identity hits to hypothetical proteins from *Aureococcus anophagefferens* (NCBI: EGB11506.1; 31% identity; e-value, 2e-21) and from *Chlorella variablis* (NCBI: EFN56803.1; 24% identity; e-value, 7e-11).

The 1.25 fold change bin was used for the identification of candidate reference genes as it offered a larger selection than the 1.10 fold change bin without including genes with increased deviations from the mean, as was the case with the 1.50 fold change bin. Thus, the 1.25 fold change category was the focus of the rest of the analyses (**Data Sheet 3**). Genes in the 1.25 fold change bin showed a broad range of mean normalized tag counts ranging from 7 to over 1200 tpm with a median of 41.94 tpm, providing for the selection of genes with different levels of constitutive expression in the cell (Figure 1). Notably, the median of the average tag counts of the genes in the ASC 1.25 fold change bin was 41.94 tpm, which is much higher than that of both Cluster 9 and Cluster 14 with median values of 14.18 tpm and 21.93 tpm, respectively.

Underlying differences in the magnitude and pattern of expression variation across treatments were identified by examining the average tag count change for each reference gene detection method (Figure 2). If all genes in a group were perfectly constitutively expressed, the average change in tag count relative to the mean observed would be 0 tpm (e.g., the tpm values across all treatments for each of the genes within a group were the same). The average variation from the mean observed in the Literature (ranging from −25.34 to 23.84 tpm) highlighted the differential expression across treatments. The average change in tag count relative to the mean in both Cluster 9 (ranging from −16.56 to 8.47) and Cluster 14 (ranging from −18.72 to 11.11 tpm) clearly demonstrated patterns of regulation across treatments (e.g., the up-regulation under P-limitation and down-regulation under Co-limited observed in Cluster 14). In contrast, the average change in tag count relative to the mean observed in the genes identified through ASC (1.25 fold change with post-*p* < 0.1), which showed a low magnitude of variation (ranging from −1.732 to 1.613 tpm) and a small mean standard deviation across the four treatments (14.24 tpm). Ultimately, the expression patterns of the majority of the genes identified through literature-based searches and *k*-means clustering were more variable across the *T. pseudonana* test treatments, than those genes identified with ASC.

**Figure 3:**
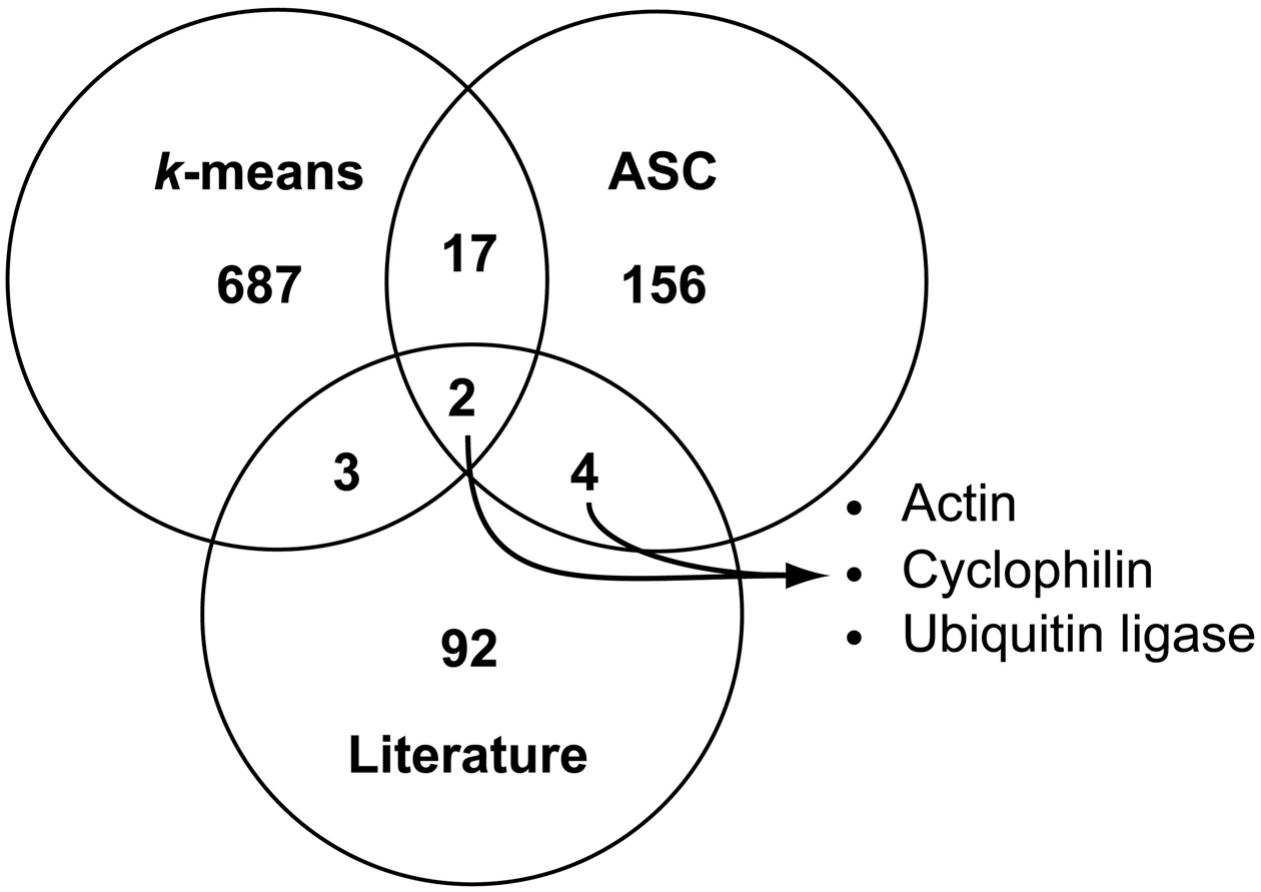
**Comparison of possible reference genes found with literature-based searches, *k*-means clustering, and ASC analysis of 1.25 fold change.** Venn diagram analysis was used to compare genes identified as candidate reference genes through literature-based homolog searches (totaling 101 genes), with the *k*-means clustering method (genes in Cluster 9 and Cluster 14, totaling 709 genes), and with quantitative exclusion by ASC (based on genes demonstrating a 1.25 fold change with a post-*p* < 0.1, totaling 179 genes). The number of genes in each region is reported. The intersection of all ASC and literature-based searches yielded six total genes representing three different gene families: actin (NCBI: 7449411), cyclophilin (NCBI: 7445376), and ubiquitin ligase (NCBI: 7448637, 7450639, 7446724, and 7451971).

A comparison of the three techniques: literature-based searches, *k*-means cluster selection, and ASC cutoff at 1.25 fold change revealed comparatively few genes in common between the techniques (Figure 3). Of the 709 genes identified through *k*-means clustering and the 179 genes found through ASC analysis (genes which pass the 1.25 fold change cutoff for post-*p* < 0.1), 21 genes are shared (Figure 3), of which six lacked GO annotations or KOG definitions (**Data Sheet 2**; **Data Sheet 3**). Between the genes identified through literature and ASC analysis, six genes were held in common; these genes were representative of the general gene classifications: actin (NCBI: 7449411), cyclophilin (NCBI: 7445376), and ubiquitin ligases (NCBI: 7448637, 7450639, 7446724, and 7451971). Only two genes (NCBI: 7448637 and 7446724) were found in common amongst all three methods of reference gene selection, both of which were annotated as putative ubiquitin ligases (**Data Sheet 1**).

## Discussion

Prior to the availability of high-throughput molecular datasets, reference genes for non-model organisms were selected based on literature reports of stably expressed genes in model organisms. With non-model organisms such as eukaryotic phytoplankton this task is particularly difficult, as stably expressed genes are not readily apparent in the relatively limited molecular literature specific to these organisms. Often the selection of a reference gene relies on information from distantly related organisms under dissimilar conditions, leading to extensive validation work (McDonald et al., 2010; Whitney et al., 2011). Herein, we compared the efficacy of reference gene selection based on the literature as compared to verifiable selection through *k*-means clustering and ASC analysis of high-throughput transcriptome data in *T. pseudonana* across four nutrient treatments (replete, P-limited, Fe-limited, and Co-limited). These treatments are of environmental relevance as both P and Fe are major drivers of diatom physiological ecology and consequently carbon fixation (Moore et al., 2004). Additionally, P and Fe often occur concurrently at very low concentrations in marine systems and have been found to be independently co-limited, or mutually exclusive biochemically (Saito et al., 2008).

Our literature-based search of relative gene expression studies from 12 algae and plants yielded 18 general reference gene categories, for which 101 homologs in the *T. pseudonana* genome were identified (**Data Sheet 1**). While some of these genes demonstrated stable expression (e.g., actin, cyclophilin, and ubiquitin conjugating enzymes), the vast majority displayed some form of differential expression in the treatments examined herein. Furthermore, there was considerable heterogeneity of expression among the different gene copies of actin, cyclophilin, and ubiquitin conjugating enzymes, demonstrating that not all genes within a gene family are stably expressed. These data underscore that a literature-based selection of reference genes necessitates validation across all treatments of interest (Vandesompele et al., 2002; Pfaffl et al., 2004).

Differential expression patterns in high-throughput datasets are often analyzed with clustering methods, such as hierarchical or *k*-means clustering (D'haeseleer, 2005). Rather than using a clustering method for the identification of differential expression patterns, here it is applied to identify constitutively expressed genes. The *k*-means clustering algorithm was chosen as it is a top–down or partition-based approach to gene clustering that is not hierarchical and requires few assumptions about the data (Hartigan and Wong, 1979). Several of the 709 putative reference genes identified by *k*-means analysis (from Clusters 9 and 14) were clearly differentially regulated, with large deviations from the mean expression level. The presence of outliers is to be expected using the *k*-means method, for it is a pattern-based method and all genes must be placed into one of the partitioned *k* = 15 clusters. Thus, optimal placement of a gene is not always guaranteed, as with a finite number of clusters, the assignment of a gene is often forced. For example, even genes in Cluster 9 and Cluster 14 were subject to strong patterns of regulation, with both clusters demonstrating large average changes in tag count relative to the mean tag count. Arguably, it is better to select a reference gene from a pool of genes that do not share the same pattern of regulation. Therefore, genes uncovered via *k*-means clustering must be manually surveyed to exclude genes with large deviation prior to the selection of a candidate reference gene.

In lieu of clustering approaches, other studies have used statistical parsing of ESTs in tomato plants (Coker and Davies, 2003) and Affymetrix whole-genome GeneChip data from *A. thaliana* (Czechowski et al., 2005) and humans (de Jonge et al., 2007) to identify reference genes that have small deviations from the mean of replicated treatments. In contrast to these and other statistical methodologies typically applied to high-throughput sequence data with replication, the Bayesian approach to gene expression analysis, ASC, allowed for selection of candidate genes based on a statistical cutoff rather than cardinality. Though typically used for the identification of differentially expressed genes, the function of ASC was reversed in this study by lowering the post-*p* cutoff. Genes for which post-*p* < 0.1 for a specified fold change were targeted, meaning that genes that were unlikely to have made that fold change were selected. The 1.25 fold change bin yielded the most options for candidate reference genes without sacrificing stability of expression (as was seen in the 1.50 fold change bin).

ASC provides a method of identifying reference genes with expression levels similar to those of target genes. For example, the mean normalized tag counts of genes identified using ASC were broad (from 7 to over 1200 tpm), providing the opportunity for reference gene expression to be generally matched with target gene expression. Current studies frequently employ reference genes for endogenous control that have very high levels of expression across all treatments, such as ACT1 (NCBI: 7449411) in *T. pseudonana* (which has a mean expression value of 1024.1 tpm in this data set), yet these highly expressed genes might not be optimal for studies of genes with low levels of expression or when multiplexing targets in probe-based RT-qPCR analysis.

High-throughput transcript datasets also allow the selection of reference genes to move beyond the confines of gene annotation and previously identified reference genes. In fact, the two genes with the most stable expression in the 1.10 fold change bin are hypothetical, with no clear annotation. Of the 179 genes that passed the 1.25 fold change cutoff with ASC, 44 lacked both GO and KOG annotations. A large percentage of the 11,390 genes in the *T. pseudonana* genome are annotated as hypothetical proteins (Armbrust et al., 2004; Mock et al., 2008), and here we show a number of them are stably expressed across the target conditions. This has been seen with model organisms, where a good majority of constitutively expressed genes fall outside the bounds of preconceived “housekeeping genes” (Czechowski et al., 2005; de Jonge et al., 2007). By using a Bayesian approach such as ASC, hypothetical proteins can be chosen as reference genes.

Comparison of the putative reference genes recovered using ASC to previous studies served to cross-validate the ASC approach. Actin (ACT1, NCBI: 7449411) has been validated in the literature as a suitable reference gene for relative expression studies of *T. pseudonana* under Fe-limitation (Whitney et al., 2011), a treatment considered in this study, and was one of the 179 genes passing the ASC 1.25 fold change cutoff. Additionally, only 5 of the 179 genes with stable expression found with ASC were identified as differentially expressed in a study of *T. pseudonana* under additional treatments to those described here (e.g., nitrogen limitation, silica limitation, etc.) (Mock et al., 2008) (**Data Sheet 4**). Of the five, only one gene (NCBI: 7451974) was identified as differentially expressed under Fe-limitation, a condition examined in this study. Taken together, this validates the genes identified with ASC using alternative data and methods, and suggests that the ASC-detected genes are globally stable across many different conditions for *T. pseudonana*. However, one of the two genes identified in the 1.10 fold change bin (NCBI: 7446346) was identified as significantly down-regulated under nitrogen limitation by Mock et al. (2008). This highlights the importance of validating genes across all treatments of interest prior to their use as reference genes.

Notably, the *k*-means and ASC dataset revealed only 21 genes in common. The 179 genes found through ASC were, in fact, distributed fairly evenly across all of the 15 clusters. The lack of intersection observed between the two datasets is likely related to the parsing ability inherent in *k*-means clustering. The *k*-means approach is highly driven by patterns of differential regulation, but does not consider the “significance” of that regulation (e.g., genes that are not significantly up-regulated are placed in a cluster with genes that are significantly up-regulated). Thus, the stably expressed genes that were identified by ASC, though not displaying major patterns of regulation, were clustered based on minor patterns in variation of gene expression. Therefore, while *k*-means clustering provides a global view of commonalities in gene expression patterns, ASC is more robust at identifying reference genes.

Eight genes were common between the ASC and literature-based searches, which were distributed across three general gene classes: actin (NCBI: 7449411), cyclophilin (NCBI: 7445376), and ubiquitin ligases (NCBI: 7448637, 7450639, 7446724, and 7451971). For those interested in identifying suitable reference genes for studies in *T. pseudonana*, but lack transcriptome datasets across the treatments of interest, these eight genes may serve as good tentative reference genes as they are verified in this study and have been identified as stable in many other organisms under many conditions. In particular, ubiquitin ligases/conjugating enzymes have been used as reference genes in several studies involving other algae, namely, *Aureococcus anophagefferens, Phaeodactylum tricornutum*, and *Prorocentrum minimum* (Siaut et al., 2007; Berg et al., 2008; McGinn and Morel, 2008a; Guo and Ki, 2011; Wurch et al., 2011), and with further analysis may represent particularly good reference genes in the phytoplankton.

Sequence-based transcriptome profiling has become an increasingly useful method for gene discovery and differential expression analysis. Yet, RT-qPCR is still valuable for the examination of detailed trends in expression in both culture and field studies. Here we show that the application of ASC and, to a lesser extent, *k*-means clustering can be used to successfully screen transcriptome data for potential reference genes. The isolation of candidate reference genes using ASC with the 1.25 fold change cutoff for post-*p* < 0.1 was more robust and stringent at excluding differentially expressed genes than both the literature-based searches and *k*-means clustering. Based on these data for *T. pseudonana*, it was shown that ACT 1 and ubiquitin ligase may be useful reference genes. Yet, in addition to these common reference genes, the data demonstrate that there are many more stably expressed genes (both annotated and hypothetical) to choose from for expression studies in this and potentially other diatoms. Notably, this survey focused only on variation in P and Fe supply, so these genes may not transfer to studies of other nutritional drivers or other physical forces, such as light intensity or temperature. As more transcriptome data are generated for phytoplankton, ASC can be employed without sequence replicates, to identify reference genes for other phytoplankton under various conditions. Additionally, the suite of genes identified through these analyses might allow for better multi-gene normalization analysis that would provide for the detection of smaller fold changes with certainty (Vandesompele et al., 2002; Czechowski et al., 2005).

## Supplementary material

The Supplementary Material for this article can be found online at: http://www.frontiersin.org/Aquatic_Microbiology/10.3389/fmicb.2012.00385/abstract

**Data Sheet 1 | Genes in the *T. pseudonana* genome homologous to reference genes from relative expression studies in algae and plants.**

**Data Sheet 2 | Putative reference genes identified with k-means clustering analysis (Cluster 9 and Clusters 14).**

**Data Sheet 3 | Putative reference genes identified with ASC analysis (*p* < 0.1 for a fold change of 1.25).**

**Data Sheet 4 | The intersection of differentially expressed genes identified by Mock et al. (2008) and stably expressed genes identified through ASC (1.25 fold change bin, *p* < 0.1).**

### Conflict of interest statement

The authors declare that the research was conducted in the absence of any commercial or financial relationships that could be construed as a potential conflict of interest.
